# Deciphering the mysteries of MEG3 LncRNA and its implications in genitourinary cancers

**DOI:** 10.3389/fonc.2025.1519103

**Published:** 2025-04-02

**Authors:** Reem A. Assal, Hannah H. Rashwan, Zeina I. Zakaria, Jana H. Sweillam, Yasmine M. Fouda, Abdelhamid M. Abdelhamid, Rana A. Youness

**Affiliations:** ^1^ Department of Pharmacology and Toxicology, Heliopolis University for Sustainable Development (HU), Cairo, Egypt; ^2^ Bioinformatics Group, Center for Informatics Science (CIS), School of Information Technology and Computer Science (ITCS), Nile University, Giza, Egypt; ^3^ Faculty of Biology, Ludwig-Maximilians-University of Munich, Munich, Germany; ^4^ Molecular Biology and Biochemistry Department, Molecular Genetics Research Team (MGRT), Faculty of Biotechnology, German International University, Cairo, Egypt; ^5^ Faculty of Medicine, Al-Kasr Al Ainy, Cairo University, Cairo, Egypt; ^6^ Biotechnology School, Nile University, Giza, Egypt

**Keywords:** LncRNA - long noncoding RNA, MEG3 lncRNA, genitourinary cancers, renal cell carcinoma, bladder cancer, prostate cancer, testicular cancer, cervical cancer

## Abstract

Maternally expressed gene 3 (MEG3), a long non-coding RNA, plays a pivotal role in various biological processes, including tumorigenesis. Aberrant expression of MEG3 has been implicated in several cancers, including genitourinary malignancies. This comprehensive review explores the multifaceted functions of MEG3 in the context of genitourinary cancers through unravelling the molecular mechanisms underlying the influence of MEG3 on cellular proliferation, apoptosis, invasion, and metastasis. Additionally, we discuss the potential clinical implications of MEG3 as a biomarker and therapeutic target in genitourinary cancers. By unraveling the intricate role of MEG3 in these biological processes, this review aims to contribute to the development of novel strategies for the diagnosis and treatment of genitourinary malignancies.

## 1 Introduction

Long non-coding RNAs (lncRNAs) are non-protein coding RNA molecules composed of more than 200 nucleotides (1, 2). However, recent discoveries have identified rare exceptions where lncRNAs encode small peptides (2–5). LncRNAs are key players in cancer development and have been validated as diagnostic and prognostic biomarkers in multiple cancers (1, 6–10). The general mechanisms by which lncRNAs act at the cellular level include signaling, scaffolding, decoying, and guiding (11–14). Although most lncRNAs exert their effects through a combination of mechanisms, the functions of lncRNAs can be broadly categorized into three main levels, namely the epigenetic, transcriptional, and post-transcriptional levels (15–18).

First, lncRNAs play a role in the epigenetic regulation of various genes (12, 19). This includes chromatin remodeling by affecting chromatin structure and regulating gene expression, DNA methylation with subsequent downregulation in target gene expression, and histone modification which could silence target genomic regions (20–22).

Second, lncRNAs could regulate gene expression on the nuclear or transcriptional level by interacting with transcription factors acting as scaffolds to prevent their binding to their target genes (22, 23). Such interactions alter the nuclear architecture through the formation of nuclear bodies such as paraspeckles and nuclear speckles which are involved in RNA metabolism and modification of splicing factors, respectively (24). Moreover, such interactions might affect pre-mRNA splicing by interacting with splicing factors, either by acting as decoys to sequester splicing factors or by inducing the phosphorylation of these factors (25–27).

Third, lncRNAs could alter the gene expression on the cytoplasmic or post transcriptional level by affecting the RNA stability and translation, binding with miRNAs to form a sponge RNA that competes with competing endogenous RNA (ceRNAs) (28–31). Such ceRNAs prevent the sponged miRNAs from performing their function or interacting with their respective target proteins (32–34).

Physiologically, the lncRNA activity is tightly regulated (35, 36). However, the dysregulation of lncRNAs in tumors worsens cancer progression and prognosis by interfering with all the reported hallmarks of cancer until now (25, 37, 38).

## 2 Structure and function of MEG3

### 2.1 Structural characteristics and genomic location of MEG3 imprint gene

**Figure 1:**
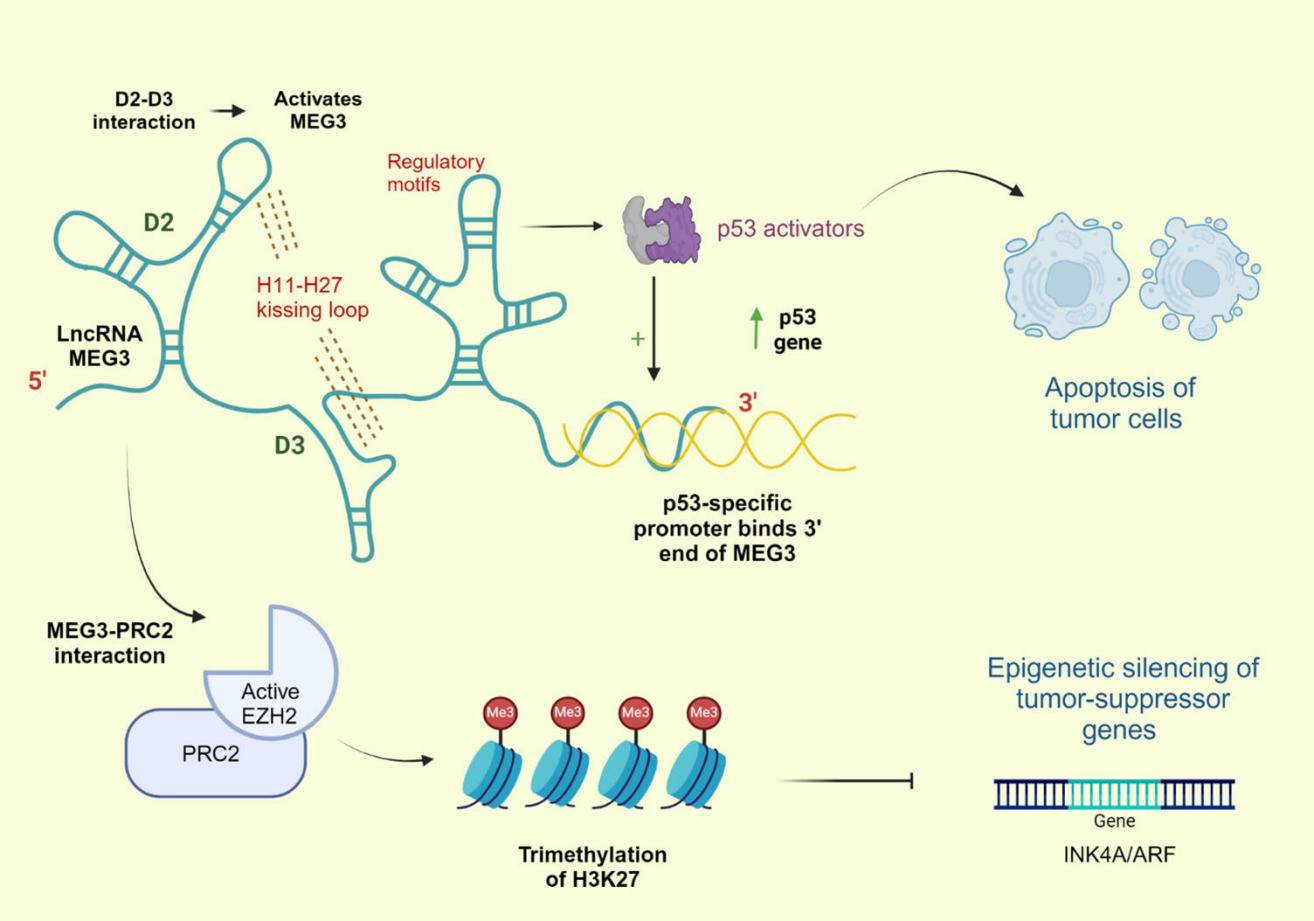
Structure and Function of MEG3. This figure illustrates the structure and function of long non-coding RNA (lncRNA) MEG3. The H11-H27 kissing loop activates MEG3 and the regulatory motifs play a role in activating p53. MEG3's interaction with p53 promotes apoptosis in tumor cells. Additionally, MEG3 interacts with PRC2 (via active EZH2), leading to the trimethylation of H3K27, which results in the epigenetic silencing of tumor-suppressor genes, such as INK4A/ARF.

The lncRNA human maternally expressed gene 3 (MEG3), an imprinted gene located on chromosome 14q32.3 within the Delta-like 1-MEG3 locus (39, 40), is exclusively maternally expressed (41, 42). Furthermore, the absence of a Kozak consensus sequence in the undefined open reading frame classifies it as a non-coding RNA (43, 44). MEG3 transcription is orchestrated by RNA polymerase 2, resulting in the splicing of the gene into 10 exons that encompass five distinct structural motifs, namely M-I to M-V (41, 45, 46). In its mature state, the 1.6 kb MEG3 transcript exhibits polyadenylation at its 3’ end and exists in both the nucleus and cytoplasm (47–49) (**Figure 1**).

### 2.2 Transcriptional regulation of MEG3

The regulation of gene expression in the Delta-like 1-MEG3 region is governed by two differentially methylated regions (DMRs) consisting of multiple methylated CpG sites: the intergenic DMR, positioned approximately 13 kb upstream from the transcription start site of MEG3, and the post-fertilization-derived secondary DMR (MEG3-DMR), which overlaps with the promoter 1.5 kb upstream (40, 42). The alternative RNA splicing process of MEG3 results in the production of approximately 12 distinct transcript isoforms within exons 1–4 and 8–10. Within this set of isoforms, variation 1 emerges as the predominant transcript. The selective expression pattern of MEG3 isoforms is tissue- and cell type-specific. All twelve MEG3 isoforms are expressed in the liver of human fetuses, while five specific isoforms, namely MEG3, MEG3b, MEG3d, MEG3e, and MEG3g, are expressed in the tissues of the pituitary gland (42, 50).

### 2.3 Physiological and pathological roles of LncRNA MEG3

Owing to its genomic location and complex molecular interplay, MEG3 plays a pivotal regulatory role in the processes of development and growth (40, 51). In addition, MEG3 plays a role in a wide variety of cellular processes, such as differentiation of osteogenic tissue and progression of bone-related conditions such as osteosarcoma, osteoarthritis, and osteoporosis (52, 53). MEG3 is highly expressed in various normal human tissues, particularly the brain and pituitary gland, especially in the gonadotrophin-producing cells (44, 54). Moreover, MEG3 has been implicated in several diseases, including ischemic neuronal death, atherosclerosis, and type II diabetes mellitus (49, 55–57). Its oncological relevance was first unveiled by Zhang et al., who revealed that MEG3 expression is deficient in pituitary adenomas, and observed that its ectopic expression resulted in the suppression of tumor cell proliferation (49, 54). Recent studies have intriguingly demonstrated a reduction in the MEG3 expression levels in various tumors, where it functions as a tumor suppressor through p53-dependent and p53-independent pathways (58, 59) (**Figure 1**).

**Figure 2:**
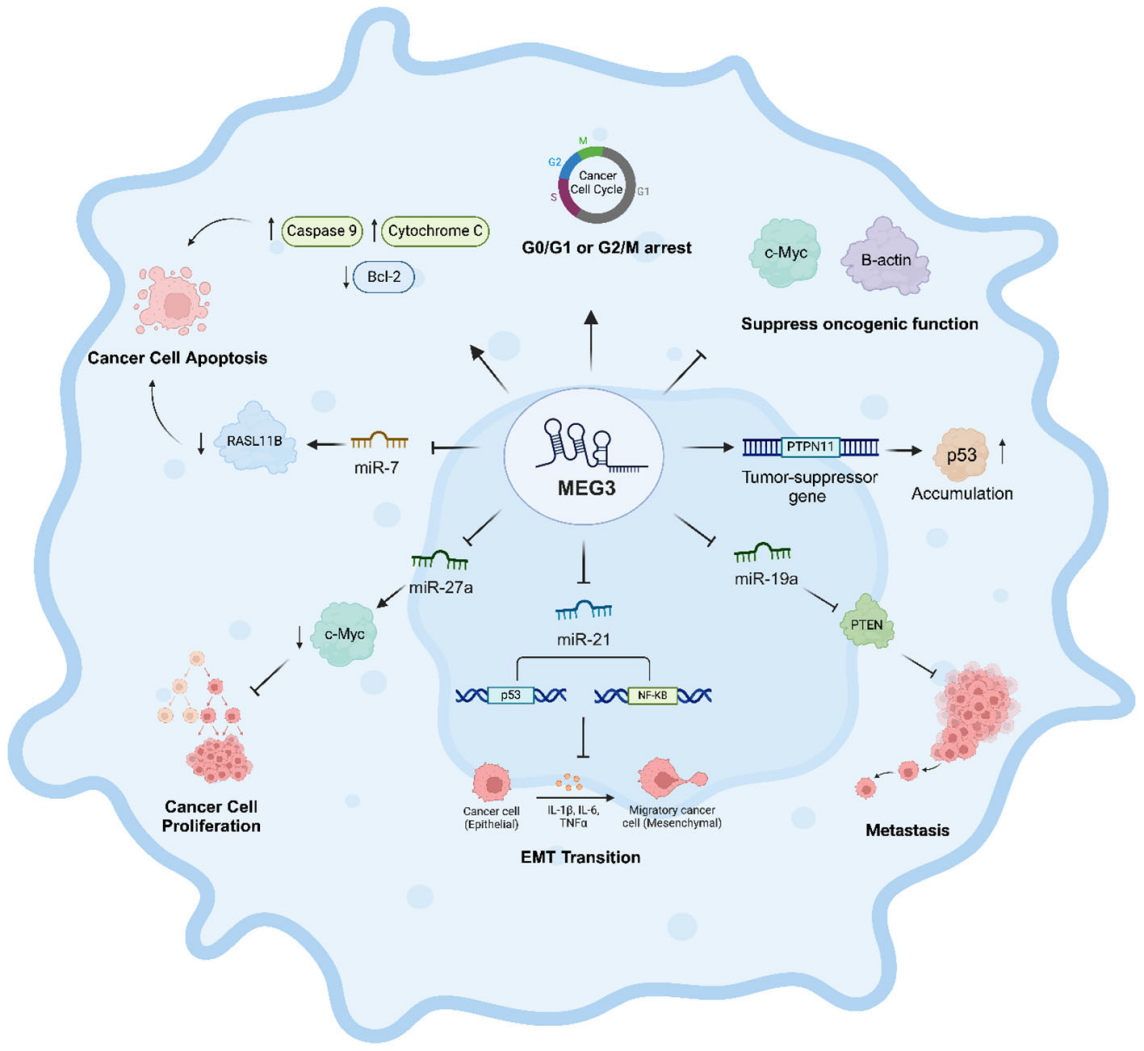
Regulatory role of MEG3 in cancer progression and suppression. This figure depicts the multifaceted tumor-suppressive role of MEG3 in cancer regulation. MEG3 induces cell cycle arrest at the G0/G1 or G2/M phases, restricting uncontrolled proliferation, and promotes apoptosis by upregulating Caspase 9 and Cytochrome C while downregulating the anti-apoptotic protein Bcl-2. It suppresses oncogenic activity by inhibiting c-Myc and B-actin and enhances tumor suppression by upregulating PTPN11, leading to increased p53 accumulation. MEG3 also inhibits metastasis by enhancing the expression of PTEN and modulates key microRNAs (miR-7, miR-27a, miR-19a, and miR-21) to balance oncogenic and tumor-suppressive pathways. Furthermore, it regulates epithelial-mesenchymal transition (EMT) by modulating p53 and NF-κB signaling, preventing the transition of epithelial cancer cells into migratory mesenchymal cells. Through these mechanisms, MEG3 acts as a crucial regulator of cancer progression and suppression.

*MEG3*, as a critical tumor suppressor gene, has the paramount potential to orchestrate many cancer hallmarks. Specifically, it can impede tumor cell proliferation, trigger apoptosis, inhibit invasion and metastasis, hinder angiogenesis, and suppress metabolic reprogramming in tumor cells (49). For instance, MEG3 demonstrated the ability to suppress the oncogenic activity of the proteins c-Myc and β-catenin in liver cancer (58). It also induces G0/G1 cell cycle arrest in prostate cancer cells (60) and G2/M arrest in cervical cancer cells (**Figure 2**) (61). Restoration of MEG3 expression suppresses tumor growth (54) and induces apoptosis in several human cancer cell lines, such as lung cancer A549 cells (62); SCC-15 and CAL27 tongue squamous cell carcinoma cell lines (63); and gastric cancer cell lines SGC7901, AGS, MGC803, MKN45, and BGC823 (59, 64).

MEG3 contains microRNA response elements, which enable MEG3 to function as a ceRNA and effectively sponge and sequester miRNAs that engage with diverse genes, proteins, such as p53, enhancer of zeste homologue 2, and nuclear factor-kappa B (**Figure 2**) (49). Through this mechanism, MEG3 can inhibit the epithelial-mesenchymal transition and metastatic potential of gastric cancer cells by sponging miR-21. This results in increased expression of the epithelial marker E-cadherin and reduced levels of mesenchymal markers, including N-cadherin, Snail, and β-catenin, along with migration-related proteins such as matrix metalloproteinase-2 (MMP-2), MMP-3, and MMP-9 (**Figure 2**) (42, 65). Furthermore, by sponging miR-19a, MEG3 modulates PTEN expression, thereby inhibiting the migratory and invasive capabilities of glioma cells (**Figure 2**) (49, 66).

Beyond its role as a ceRNA, MEG3 can influence gene activity through mechanisms such as translation, transcription, post-translational modifications, and epigenetic regulation. Several studies have linked MEG3 dysregulation to poor clinical outcomes and the development of drug resistance. For instance, MEG3 may ameliorate p53 levels by augmenting its transcriptional activity and post-translational modifications (49). It can also decrease the levels of murine double minute 2, an E3 ubiquitin ligase that facilitates p53 ubiquitination and proteasomal degradation, leading to the stabilization of p53 protein and activation of its downstream targets (49, 67). Additionally, MEG3 inhibition has been associated with nickel-induced hypermethylation through increased DNMT3b levels, while the suppression of PHLPP1 transcription has been attributed to decreased interaction between MEG3 and its repressive transcription partner c-Jun (53, 68). Moreover, upregulation of MEG3 expression increases the sensitization of tumor cells to radiation therapy and chemotherapy, thereby boosting the efficacy of current treatment approaches (50). Nevertheless, the functional properties of MEG3 and its involvement in both physiological and pathologic cellular processes are still under active investigation.

## 3 Deep comparison between the role of MEG3 and other lncRNAs

Upon examining the tumour-suppressive roles of lncRNAs in genitourinary cancers, MEG3 has emerged as a notable candidate. It is consistently downregulated in tumor tissues compared to normal ones and exhibits significant tumor suppressive roles across all genitourinary cancers. MEG3 primarily functions by promoting apoptosis and by inhibiting proliferation, angiogenesis and metastasis, marking it as a universal tumor suppressor in these cancers.

Among other tumour-suppressive lncRNAs, GAS5 stands out as most similar to MEG3. GAS5 has been identified as a robust tumor suppressor in several genitourinary cancers, including ovarian, RCC, bladder, prostate, and cervical cancers. In ovarian cancer, GAS5 disrupts mitochondrial membrane potential and influences apoptosis pathways, similarly to MEG3, through the promotion of BAX, BAK, and caspases (69, 70). Additionally, it regulates cell proliferation by inhibiting miR-21, thereby increasing SPRY2 expression, crucial for suppressing tumor growth and proliferation (71).

In RCC, GAS5 shares functions with MEG3 by inhibiting cell proliferation, migration, invasion, and inducing apoptosis and cell cycle arrest (**Table 1**) (72). In bladder cancer, while GAS5 suppresses cell proliferation by modulating cellular components like CCL1, it complements MEG3’s actions by also targeting major oncogenic pathways (73). In prostate cancer, GAS5 functions by inducing apoptosis and repressing androgen receptor (AR) activity, sequestering the androgen/AR complex and inhibiting the AR signaling pathway, which is vital for prostate cancer cell survival and growth (74, 75). Unlike MEG3, GAS5’s mechanism primarily focuses on disrupting hormone-driven growth signals. Furthermore, in cervical cancer, GAS5 acts as a growth and metastasis inhibitor by directly binding and downregulating miR-196a and miR-205, crucial for the proliferation, invasion, and apoptosis of cervical cancer cells (**Table 1**) (76, 77). GAS5’s broad impact on tumor suppression through miRNA regulation parallels MEG3’s function, though it targets different specific miRNAs, enhancing the collective miRNA regulatory spectrum in this cancer type.

**Table 1:** Illustrates the comparison between the role of MEG3 and other lncRNAs.

LncRNA	Cancer Types	Mechanism of Action	Effects on Tumour	Regulation	References
GAS5	Ovarian, RCC, bladder, prostate, cervical	Disrupts mitochondrial potential, regulates apoptosis, inhibits proliferation	Suppresses proliferation, migration, invasion, apoptosis	Tumor suppressor	(69–77)
MAGI2-AS3	Ovarian, bladder	Sponges miRNAs, inhibits MYC signaling, affects cell migration and proliferation	Reduces metastasis and migration, affects oncogenic pathways	Tumor suppressor	(78–80)
CASC2	RCC, bladder	Inhibits miR-21, regulates Wnt/β-catenin, promotes cell death	Reduces proliferation, migration, and invasion	Tumor suppressor	(81, 82)
PTENP1	Bladder, cervical	Enhances expression of tumor suppressors (e.g., PDCD4, PTEN) via miRNA sequestration	Inhibits growth, migration, metastasis, and EMT	Tumor suppressor	(83–85)
TUSC8	Cervical	Inhibits invasion and migration via miR-641/PTEN axis	Reduces invasiveness, promotes apoptosis	Tumor suppressor	(86)
HAND2-AS1	Ovarian	Reduces metastatic potential by regulating cell adhesion, migration	Suppresses metastasis, reduces viability	Tumor suppressor	(87)
HOXA11-AS	Ovarian	Modulates transcriptional pathways, likely in EMT regulation	Reduces metastasis	Tumor suppressor	(88)
HOTAIRM1	Ovarian	Suppresses proliferation, invasion via miRNA sponging	Reduces metastasis, affects ARHGAP24	Tumor suppressor	(89)
CASC2a	Endometrial	Inhibits growth via epigenetic gene inactivation	Suppresses proliferation	Tumor suppressor	(90, 91)
PCDH10	Endometrial	Inhibits cell growth, promotes apoptosis via methylation changes	Enhances apoptosis, inhibits growth	Tumor suppressor	(92)
LINC00261	Endometrial	Modulates miRNA/FOXO1 axis, affecting migration and invasion	Reduces proliferation, migration, invasion	Tumor suppressor	(93)
TUSC7 and LINC00672	Endometrial	Enhances chemosensitivity, promotes apoptosis via p53	Increases chemotherapy response, apoptosis	Tumor suppressor	(94, 95)
H19	Prostate	Represses TGFβ1, modulates metastasis via miR-675	Suppresses metastasis, reduces invasion	Tumor suppressor	(74, 96)
PCAT29	Prostate	Regulates androgen receptor, inversely correlated with proliferation	Reduces proliferation and migration	Tumor suppressor	(97)
RFPL3S	Testicular Germ Cell Tumors (TGCT)	Linked to hypermethylation, affects cell invasion and proliferation	Inhibits invasion and proliferation	Tumor suppressor	(98)
SARCC	RCC	Targets VHL-mutant RCC cells, reduces AR expression, affects HIF-2α and C-MYC	Inhibits proliferation, migration, invasion	Tumor suppressor	(99)
NBAT1	RCC	Inhibits proliferation, migration, invasion	Inhibits tumor growth, serves as a prognostic biomarker	Tumor suppressor	(100)
BX357664	RCC	Modulates EMT, affects MMP2, MMP9, TGF-β1/p38/HSP27 signaling	Suppresses proliferation, migration, invasion	Tumor suppressor	(101)
BANCR	Bladder	Increases apoptosis, reduces migration and proliferation	Promotes apoptosis, inhibits migration	Tumor suppressor	(102)
MDC1-AS	Bladder	Inhibits malignant phenotype via MDC1 upregulation	Reduces proliferation, migration, invasion	Tumor suppressor	(103)
LINC00312 and LINC00641	Bladder	Sponges miR-197-3p, affects migration and invasion	Inhibits migration and invasion	Tumor suppressor	(104, 105)
MEG3	Genitourinary (ovarian, RCC, bladder, prostate, cervical)	Promotes apoptosis, inhibits proliferation, angiogenesis, metastasis	Universal tumor suppressor, inhibits growth and metastasis	Tumor suppressor	(106, 107) (108–115)

The lncRNA MAGI2-AS3 exhibits tumour-suppressive functions in various cancers by sponging multiple miRNAs, including miR-15-5p, miR-374a-5p, miR-374b-5p, and miR-525-5p. In ovarian cancer, it inhibits MYC signaling, significantly reducing cell proliferation and migration, illustrating its capacity to suppress multiple oncogenic pathways (**Table 1**) (78, 79). This broad-spectrum targeting complements MEG3’s more focused approach against specific miRNAs and pathways like YBX1 and EGFR. In bladder cancer, MAGI2-AS3 operates through a ceRNA mechanism involving the MAGI2-AS3/miR-31–5p/TNS1 axis, with its downregulation linked to increased migration, proliferation, and invasion, emphasizing its role in metastasis and tumor staging (**Table 1**) (80).

Another lncRNA, CASC2, acts as a tumor suppressor in RCC by targeting miR-21, which leads to reduced proliferation and migration (81). Its effect on miR-21 provides a specific focal point that complements MEG3’s broader regulatory effects. In bladder cancer, CASC2 modulates the Wnt/β-catenin signaling pathway and promotes cell death, significantly impacting proliferation, migration, and invasion (**Table 1**) (82). These activities align with MEG3’s, presenting a coordinated defense against key signaling pathways that promote tumor growth. Moreover, PTENP1, functioning analogously to MEG3 in bladder cancer, enhances the expression of tumor suppressors like PDCD4 by suppressing miR-20a, thereby inhibiting tumor growth and metastasis (**Table 1**) (83).

In cervical cancer, PTENP1 targets key regulatory pathways by enhancing the expression of the tumor suppressor PTEN through the sequestration of miR-106b, inhibiting cell growth, motility, and EMT, crucial factors in cancer progression and metastasis (**Table 1**) (84, 85). Another lncRNA, TUSC8, demonstrated tumour-suppressive roles in cervical cancer where it focuses on the inhibition of invasion and migration through the miR-641/PTEN axis, similar to PTENP1, but with a specific miRNA target. By upregulating PTEN, TUSC8 curtails pathways essential for cancer cell invasiveness, supporting a role that complements both MEG3 and PTENP1 in modulating the tumor microenvironment (**Table 1**) (86).

In ovarian cancer, several lncRNAs like HAND2-AS1, HOXA11-AS, and HOTAIRM1 demonstrate tumor-suppressive roles similar to MEG3. HAND2-AS1 reduces metastatic potential by decreasing cell adhesion, migration, and viability, aligning with MEG3’s regulation of epithelial-mesenchymal transition (EMT) (87). HOXA11-AS, downregulated in epithelial ovarian cancer, likely modulates transcriptional pathways, complementing MEG3’s role in metastasis control (88). HOTAIRM1 suppresses proliferation and invasion by enhancing ARHGAP24 expression and sponging miR-106a-5p, mirroring MEG3’s anti-metastatic effects (89). In endometrial cancer, CASC2a and PCDH10 act as tumor suppressors with unique mechanisms. CASC2a, downregulated in the cancer, inhibits growth through epigenetic gene inactivation, contrasting with MEG3’s approach (90, 91).

PCDH10, re-expressed after methylation-induced downregulation, curbs growth and enhances apoptosis, paralleling MEG3’s effects but through methylation changes (92). LINC00261 also reduces proliferation, migration, and invasion by modulating the miRNA/FOXO1 axis, reflecting MEG3’s miRNA modulation strategy (93). TUSC7 and LINC00672 enhance chemotherapeutic responses in endometrial cancer, with TUSC7 increasing sensitivity to treatments like CDDP and Taxol, and LINC00672 promoting chemosensitivity through p53-mediated gene suppression. Both complement MEG3’s effects on cell cycle and apoptosis, offering synergistic benefits (**Table 1**) (94, 95).

In prostate cancer, lncRNAs such as H19 and PCAT29 play critical but distinct roles in modulating tumor behavior. H19, typically oncogenic in other cancers, serves as a tumor suppressor in metastatic prostate cancer. It specifically represses the effects of transforming growth factor beta 1 (TGFβ1), a key factor in metastasis, and modulates metastasis through the H19-miR-675 axis. This targeted regulation provides a pathway-specific suppression that complements MEG3’s broader influence on cellular apoptosis and invasion (**Table 1**) (74, 96). Additionally, PCAT29 is recognized as an androgen-regulated tumor suppressor whose expression is inversely correlated with the proliferation and migration of prostate cancer cells. This regulatory role on androgen signals offers a distinct but synergistic mechanism alongside MEG3’s modulation of epigenetic and miRNA pathways, enhancing the overall suppression of tumor growth and metastasis in prostate cancer (**Table 1**) (97).

In testicular germ cell tumors (TGCT), the lncRNA RFPL3S exhibits a significant reduction, which is associated with hypermethylation and low copy number variations. The downregulation of RFPL3S is linked to enhanced cell invasion and proliferation in TGCT, positioning it as a potential prognostic marker (**Table 1**) (98). This suggests that while MEG3 modulates miRNA interactions to affect cell survival pathways, RFPL3S’s influence primarily arises from epigenetic changes that affect its expression.

In RCC, the lncRNA SARCC targets VHL-mutant RCC cells, inhibiting proliferation by reducing the stability and expression of the androgen receptor (AR). This suppression of AR decreases HIF-2α and C-MYC expression, focusing on hormonal and hypoxia-related pathways (**Table 1**) (99). Unlike SARCC, MEG3 has a broader impact on signaling and apoptosis pathways, indicating a more extensive role in RCC tumor suppression. Additionally, NBAT1 functions as a tumor suppressor in RCC, inhibiting proliferation, migration, and invasion, aligning with MEG3’s effects and serving as a prognostic biomarker (**Table 1**) (100). In other findings, BX357664 acts as a tumor suppressor by modulating epithelial-mesenchymal transition (EMT) and affecting MMP2, MMP9, and TGF-β1/p38/HSP27 signaling. It inhibits proliferation, migration, invasion, and impacts the cell cycle, providing a complementary mechanism to MEG3’s modulation of the cell cycle and apoptosis (101).

In bladder cancer, BANCR increases apoptosis and reduces migration and proliferation when overexpressed, focusing on apoptosis rather than miRNA sponging. Its functional outcomes align with those of MEG3, making them cooperative in curbing tumor growth (102). MDC1-AS inhibits the malignant phenotype through the up-regulation of MDC1, decreasing proliferation, migration, and invasion, and supports MEG3’s effects on cell cycle and apoptosis (**Table 1**) (103). Furthermore, LINC00312 and LINC00641 target migration and invasion pathways by sponging miR-197-3p. Their approach mirrors MEG3’s strategy of miRNA modulation but targets different specific miRNAs, enhancing the suppression of invasive behavior in bladder cancer (104, 105).

## 4 The role of MEG3 in genitourinary cancers

### 4.1 Renal cell carcinoma

Renal cell cancer, also known as renal cell carcinoma (RCC), is the most prevalent form of kidney cancer in adults, accounting for approximately 90% of cases. It includes clear cell, papillary, and chromophobe subtypes. Its incidence has consistently risen over the past few decades, and it poses a significant threat to global health (116). It has a mortality rate between 30 and 40% and is more prevalent in males than in females.

The aggressiveness of RCC varies considerably between individuals and is affected by several variables. More than 60% of patients with RCC are detected incidentally during routine ultrasound examination, and only 10% of patients exhibit characteristic clinical symptoms (117). Accordingly, a significant proportion of RCC cases are diagnosed at advanced stages, when the tumor has already spread beyond the kidney, resulting in inferior outcomes and limited treatment options. Certain histological RCC subtypes, including clear cell carcinoma and papillary carcinoma, are typically associated with increased aggressiveness and metastasis risk than other subtypes (118).

**Figure 3:**
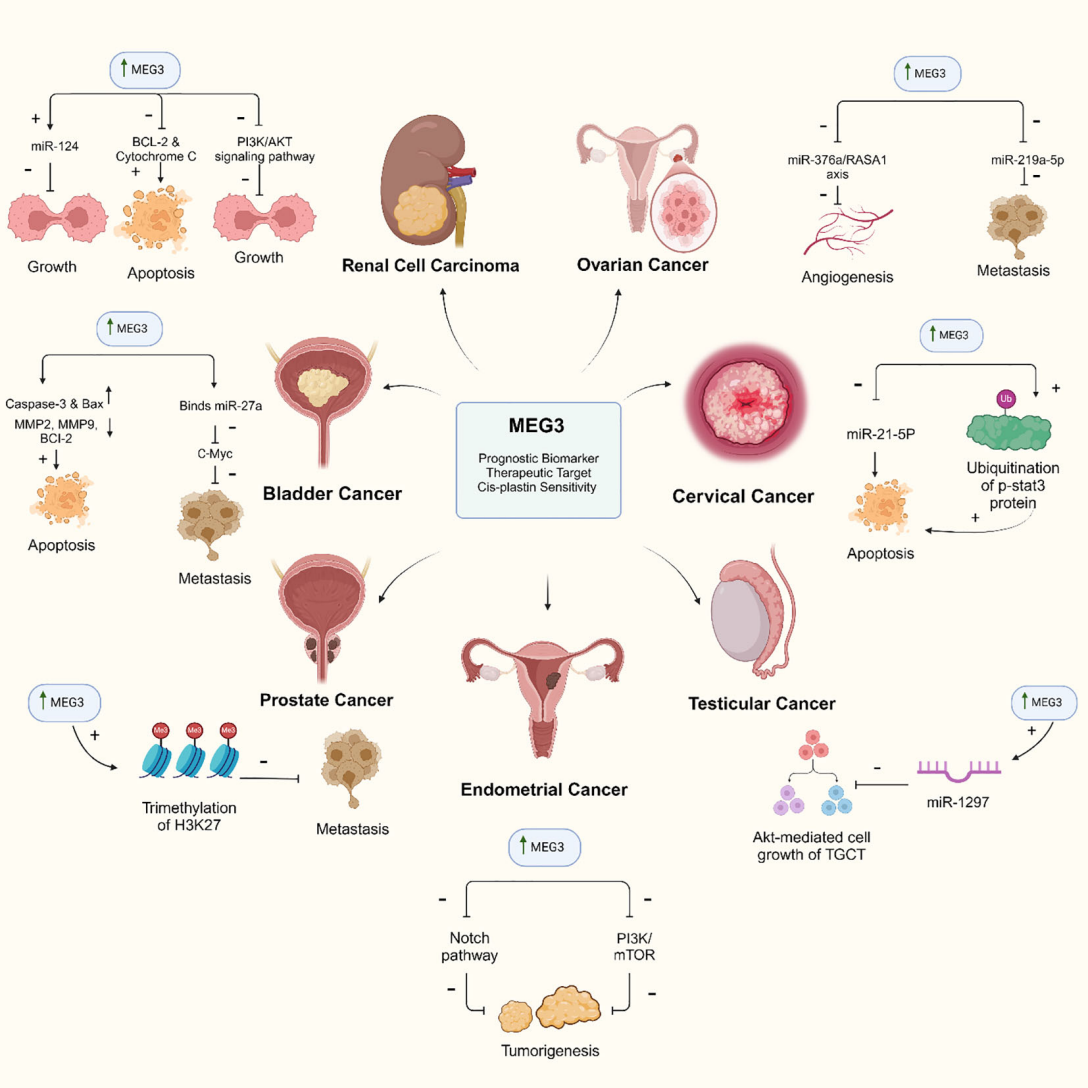
MEG3 lncRNA: a key regulator of tumor suppression across multiple cancer pathways. The diagram illustrates the role of MEG3 in various cancers as a prognostic biomarker, therapeutic target, and enhancer of cis-platin sensitivity. In renal cell carcinoma, MEG3 inhibits the PI3K/AKT signaling pathway, reducing tumor growth and promoting apoptosis. In ovarian cancer, it blocks angiogenesis and metastasis through specific miRNA pathways. MEG3 also induces apoptosis in bladder cancer and inhibits metastasis by downregulating oncogenes. In cervical cancer, MEG3 promotes apoptosis by inhibiting miR-21-5p and STAT3. It reduces cell growth in testicular cancer by regulating miR-1297. In endometrial cancer, MEG3 inhibits tumor growth by suppressing the Notch and PI3K/mTOR pathways, while in prostate cancer, it prevents metastasis through H3K27 trimethylation.

The role of MEG3 in RCC has been investigated in many studies, shedding light on its impact on cancer progression and potential therapeutic implications. MEG3 is downregulated in RCC tissues and cell lines. Its overexpression resulted in decreased proliferation, migration, and invasion of RCC cells via regulation of ST3Gal1 transcription and EGFR sialylation, thereby influencing the PI3K-AKT pathway (106). In addition, MEG3 was identified as an inducer of RCC cell apoptosis by activating the mitochondrial pathway, as indicated by decreased Bcl-2 expression and elevated levels of cleaved caspase-9 and cytochrome c protein (107) (**Figure 3**). Moreover, the combined expression of miR-124 and MEG3 emerged as an independent prognostic factor in RCC, where the overexpression of miR-124 or MEG3 inhibited cell proliferation, migration, and invasion, and restrained tumor growth. Additionally, MEG3 induced p53 protein accumulation and regulated the tumor-suppressive gene *PTPN11* (119) (**Table 2**) (**Figure 3**). MEG3 regulates miR-7/RASL11B signaling in clear cell renal cell carcinoma, thereby promoting apoptosis; inhibiting cell proliferation, migration, and invasion; and inducing G0/G1 cell cycle arrest (120). In papillary renal cell carcinoma, MEG3 expression was downregulated in tumor tissues relative to that in adjacent normal tissues (**Table 2**) (133). These findings implicate MEG3 as a potential prognostic biomarker and molecular therapeutic target for RCC management.

**Table 2:** Summarizes the functions of MEG3 across genitourinary cancers.

Tumor	MEG3 Expression	Function role	Related genes	miRNA	Biomarker type	References
Renal cell carcinoma (RCC)	Downregulated	Proliferation, migration, invasion, apoptosis	ST3Gall EGFR Bcl-2 PTPN11 RASL11B	miR-124 miR-7	Prognostic, therapeutic target	(106, 107) (119, 120)
Bladder cancer	Downregulated	Proliferation, migration, invasion,apoptosis	c-Myc PTEN MMP2 MMP9	miR-494 miR-27a	Diagnostic, prognostic and therapeutic target	(108, 109) (121)
Prostate cancer	Downregulated	Proliferation, migration, invasion, apoptosis	QKI-5 H3K27	miR-9-5p	Prognostic and therapeutic target	(110, 111)
Testicular cancer	Downregulated	Proliferation, apoptosis	PTEN	miR-1297	Therapeutic target	(11, 12)
Cervical cancer	Downregulated	Proliferation, apoptosis, invasion, migration	BTG1 STC1 PTEN P-STAT3	miR-21-5p miR-421 miR-7-5p miR-21	Diagnostic, prognostic and therapeutic target	(112, 113) (122–127)
Ovarian cancer	Downregulated	Angiogenesis, invasion, apoptosis	YBX1 RASA1 LAMA4 PTEN EGFR	miR-376a miR-30e-3p miR-219a-5p miR-205-5p	Prognostic and therapeutic target	(114, 115) (128–130)
Endometrial cancer	Downregulated	Proliferation, apoptosis, invasion, migration	MTOR PD-L1	miR-216a	Therapeutic target	(131, 132)

Furthermore, the expression of MEG3 has been studied in various types of renal cell carcinoma (RCC), including clear cell RCC (ccRCC) and papillary RCC (pRCC). In ccRCC, MEG3 expression is generally significantly reduced compared to normal kidney tissue. This downregulation of MEG3 has been associated with proliferation, migration and invasion in patients with ccRCC (120). The loss of MEG3 expression in ccRCC is thought to contribute to the pathogenesis of the disease through mechanisms such as up-regulating *RASL11B* to induce G0/G1 cell cycle arrest; and promoting cell apoptosis by suppressing miR-7 in ccRCC (120).

The expression patterns of MEG3 in pRCC are less well-defined but suggest a similar trend of downregulation compared to normal tissues (133). However, the extent and implications of MEG3 downregulation in pRCC are not as clearly established as in ccRCC. Studies suggest that the pathways and the impact of MEG3 downregulation in pRCC may differ slightly due to the histological and molecular differences between ccRCC and pRCC (118).Thus, while both ccRCC and pRCC show a trend of reduced MEG3 expression, the details, extent, and implications of this downregulation appear to vary, reflecting the distinct biological behaviors of these RCC subtypes. Further research is needed to fully elucidate the role of MEG3 in different RCC types and its potential as a therapeutic target or biomarker.

### 4.2 Bladder cancer

Bladder cancer is a common cancer with higher incidence rates in males than those in females. Trends in incidence vary by country, with stabilizing or declining rates among males and increasing rates among females. Mortality rates have been declining, especially in developed countries. The 5-year survival rate for muscle-invasive and metastatic bladder cancer is less than 5% (134).

The most common type of bladder cancer is transitional cell carcinoma. Other less common types include squamous cell carcinoma and adenocarcinoma. The extent of tumor invasion into the bladder wall and the presence of lymph node involvement or distal metastasis determines the tumor stage. The TNM staging system is commonly used to assess the stage of bladder cancer, with higher stages indicating a more aggressive disease. Molecular profiling advancements have expanded our understanding of the aggressiveness of bladder cancer. Mutations or alterations in genes such as *TP53*, *RB1*, and *FGFR3* can indicate a more aggressive tumor behavior (135).

MEG3 plays a vital role in the suppression of tumors in several forms of cancer, including bladder cancer. In bladder cancer tissues, MEG3 is significantly reduced compared to healthy controls. The fact that MEG3 levels are negatively correlated with those of the autophagy marker LC3-II suggests that MEG3 may play a role in autophagy regulation. In human bladder cancer cell lines, activation of MEG3 inhibited autophagy, whereas inhibition of MEG3 induced autophagy. The downregulation of MEG3 inhibits apoptosis and induces cellular proliferation, but these effects are reversed when autophagy is inhibited (136).

MEG3 serves a crucial role in inhibiting the invasion and metastasis of bladder cancer cells. It functions as a ceRNA by negatively modulating c-Myc through competing with PHLPP2 mRNA for binding with miR-27a. The potent proto-oncogene *c-Myc* promotes proliferation, invasion, and apoptosis in numerous malignancies. Overexpression of MEG3 inhibited the transcription of *c-Myc* in a c-Jun-dependent manner, thereby preventing bladder cancer invasion and metastasis (108) (**Figure 3**).

Overexpression of MEG3 increases the sensitivity of bladder cancer cells to the chemotherapeutic agent, cisplatin. Mutual expression of MEG3 and p53 suggests that they may participate in a positive feedback cycle. In bladder cancer cells treated with cisplatin, MEG3 overexpression induces cell apoptosis, downregulates the anti-apoptotic protein Bcl2, and upregulates the pro-apoptotic proteins cleaved-caspase-3 and Bax. Moreover, overexpression of MEG3 inhibits cell invasion and downregulates MMP2 and MMP9 in bladder cancer cells (121) (**Figure 3**, **Table 2**).

Patients with bladder cancer have lower serum levels of MEG3 than healthy subjects and patients with benign disease. Moreover, a decrease in MEG3 expression is associated with a shortened relapse-free time (137) (**Table 2**). These findings demonstrate the therapeutic potential of MEG3 to improve the efficacy of chemotherapy and inhibit the progression of bladder cancer. They also indicate that MEG3 may serve as a diagnostic and prognostic marker for bladder cancer. Additional research is required to fully elucidate the underlying mechanisms and investigate the therapeutic potential of MEG3 for the management of bladder cancer.

### 4.3 Prostate cancer

Prostate cancer ranges from indolent, slowly growing tumors with a good prognosis to aggressive, metastasizing cancers with poor outcomes. Prostate cancer aggressiveness is determined by Gleason score, PSA levels, and tumor stage. Higher Gleason scores and PSA values indicate more aggressive illness. Genetic changes and biomarkers may also affect tumor behavior. The aggressiveness of prostate cancer requires early detection and personalized treatment. Surgery, radiation, hormone therapy, and targeted drugs can slow the progression of prostate cancer and improve survival. Early diagnosis and risk classification are key to adapting effective management approaches (138).

Various non-coding RNAs are implicated in the pathogenesis of prostate cancer. Among them, MEG3 was found to be down-regulated in prostate cancer and modulated the miR-9-5p/QKI-5 axis; thus, affecting cell proliferation, migration, invasion, and apoptosis rate (110). Additionally, MEG3 inhibits prostate cancer progression by facilitating H3K27 trimethylation of EN2 by binding to enhancer of zeste homologue 2 (111) (**Figure 3**). However, both MEG3 polymorphisms, rs11627993 C>T and rs7158663 A>G, did not appear to affect prostate cancer susceptibility (**Table 2**) (139). Collectively, these studies demonstrate the diverse regulatory functions of MEG3 in prostate cancer, providing potential insights into its impact on cancer progression and facilitating the identification of novel therapeutic targets for this disease.

### 4.4 Testicular cancer

While testicular cancers can arise from various cell types, the majority are germ cell tumors. These tumors predominantly occur in young men, although they can be diagnosed at any age (140).

Despite being a rare tumor, testicular germ cell tumor (TGCT) was reported as the most commonly occurring cancer in males aged 15–44 years in the US (141). It was predicted that the occurrence of TGCT will remain on the rise with the highest incidence in Hispanics compared to that in any other racial or ethnic group in the US (142). Histologically, the TGCTs can be classified into two groups, seminomas and non-seminomas (143). While seminomas are similar to PGCs, non-seminomas are divided into three classes, undifferentiated embryonal carcinoma, extra-embryonic (yolk sac choriocarcinoma) patterning, or differentiated as a teratoma (143). The European Association of Urology predicted the relapse of 15–30% of patients with TGCT after receiving the first line of chemotherapy and the patients will require further therapies (144). Moreover, primary TGCT can metastasize to several organs including the brain, heart, lung, liver, and lymph nodes (145). In normal spermatogenesis, primordial germ cells (PGCs) develop into gonocytes, followed by multiple developmental steps to eventually maturing into haploid spermatozoa (146, 147). However, if PGCs fail to mature into gonocytes either fetal or postnatally, intratubular germ cell neoplasia, also known as carcinoma *in situ* develops (148), which is similar to PGCs and their genomes remains undifferentiated and unmethylated (149). Next, intratubular germ cell neoplasia progresses to TGCTs through the loss of PTEN and p21 and the gain of murine double minute 2 expression (150, 151). Moreover, *KRAS* mutations, which are unique to TGCT, are not present in the intratubular germ cell neoplasia stage (152). Conventionally, the treatment of the TGCTs is a combination of radiotherapy along with chemotherapy, lymph node dissection, or radical orchiectomy.

The MEG3 expression levels are markedly reduced in TGCT, while the expression levels of miR-1297 were unchanged. Moreover, PTEN protein levels were lower in the tumor tissues compared to those in the paired adjacent non-tumor tissues; however, the PTEN transcript levels were unchanged. The authors hypothesized that there is post-transcriptional control of miR-1297 on the PTEN transcripts (**Table 2**) (12). Bioinformatic analyses revealed that miR-1297 can bind both MEG3 lncRNA and PTEN mRNA in TGCT cells. Overexpressing the MEG3 in TGCT cells led to the competitive binding of MEG3 lncRNA to miR-1297 rather than PTEN, allowing PTEN to suppress the Akt-mediated cell growth of the TGCT. Thus, overexpression of MEG3 could be a valuable tool for the treatment of TGCT (11) (**Table 2**, **Figure 3**).

### 4.5 Cervical cancer

Cervical cancer remains one of the most prevalent gynecologic malignancies worldwide. It ranks fourteenth among all malignancies and fourth among females worldwide (153). Each year, more than 500,000 females are diagnosed with cervical cancer, resulting in more than 300,000 fatalities worldwide (154). Globally, the age-adjusted incidence of cervical cancer was estimated to be 13.1 per 100,000 females, with rates ranging from less than 2 to 75 per 100,000 females, depending on the country. In eastern, western, central, and southern Africa, cervical cancer was the primary cause of cancer-related deaths among females (155).

Human papillomavirus (HPV) infection, particularly HPV types 16 and 18, is a significant etiologic factor for cervical cancer. Infections with high-risk HPV can result in persistent infection with subsequent genomic instability, thereby promoting the transformation of normal cervical cells into cancerous ones (156). Moreover, changes in particular genes and molecular pathways play a crucial role in the aggressiveness of cervical cancer. Mutations or dysregulations in *TP53*, *p16INK4a*, and *PTEN* can disrupt cell cycle control and promote uncontrolled cell proliferation, migration, and invasion (157) (158). In addition, the expression levels of specific biomarkers have been linked to the aggressiveness of cervical cancer as they facilitate extracellular matrix degradation and tissue remodeling. The altered expression of MMPs have been linked to an increase in tumor invasion and metastasis (159). Furthermore, studies have highlighted the function of lncRNAs in modulating the aggressiveness of cervical cancer. Certain lncRNAs have the potential to function as oncogenes or tumor suppressors, influencing cell proliferation, migration, and invasion (160).

MEG3 is a tumor suppressor that influences the growth, proliferation, and apoptosis of cervical cancer cells by modulating multiple molecular pathways and signaling cascades. MEG3 is consistently expressed at low levels in cervical cancer tissues and cell lines. However, increasing the MEG3 levels in cervical cancer cell lines can have significant effects. Elevated MEG3 levels inhibited cell proliferation, induced cell cycle arrest, and promoted apoptosis (122). In addition, MEG3 operates as a cancer suppressor by reducing the levels of miR-21-5p in cervical cancer cell lines (112) (**Figure 3**). Notably, inhibition of MEG3 in specific cell lines, such as HeLa and CaSki cells, significantly increased the expression of the oncomiR miR-21-5p (112). This highlights the potential function of MEG3 as a tumor suppressor that is capable of inhibiting the growth of cervical cancer (161). Downregulating MEG3 in cervical cancer promoted cellular proliferation, primarily via the regulation of miR-21 (112).

Moreover, MEG3 appears to have diagnostic and prognostic value in cervical cancer. It can be used as a diagnostic marker and prognostic indicator, especially for lymph node metastasis and FIGO staging. Low MEG3 levels, lymph node metastasis, and advanced FIGO stage (III and IV) have been identified as independent cervical cancer prognostic factors (113). A previous study revealed that the lncRNA-MEG3/miR-421/BTG1 pathway modulation by lidocaine inhibited the proliferation of cervical cancer cells and induced apoptosis (**Table 2**) (123). Additionally, MEG3 induced apoptosis in cervical carcinoma cells through endoplasmic reticulum stress and the miR-7-5p/STC1 axis (124). In cervical cancer tissues and cell lines, the expression of MEG3 and STC1 is diminished, while the expression levels of miR-7-5p are elevated. Overexpression of MEG3 or inhibition of miR-7-5p induced apoptosis in cervical carcinoma cells in response to endoplasmic reticulum stress (124). Furthermore, decreased MEG3 expression appears to be an important indicator of the transition from premalignant cervical lesions to invasive cancer (125). In addition, by modulating the miR-21/PTEN axis, MEG3 promoted cisplatin sensitivity in cervical cancer cells. Conversely, knocking down MEG3 promoted cervical cancer cell proliferation, migration, and inhibited apoptosis, particularly in the presence of cisplatin (126).

Besides being a tumor suppressor, MEG3 inhibits the proliferation of cervical cancer cells by promoting the ubiquitination-mediated degradation of P-STAT3 protein. MEG3 binds to P-STAT3 in cervical cancer cells, leading to its ubiquitination and subsequent degradation, resulting in apoptosis and inhibition of tumor cell proliferation (**Table 2**, **Figure 3**). A better understanding of the regulatory axis MEG3-STAT3 in cervical cancer may provide novel insights for potential treatment strategies (127). In conclusion, owing to its significant role in cervical cancer, MEG3 could potentially act as a diagnostic, prognostic, and therapeutic target.

### 4.6 Ovarian cancer

Ovarian cancer is the seventh most prevalent cancer in females and the eighth leading cause of cancer-related mortality, with five-year survival rates below 45%. Although age-standardized rates are stable or declining in the majority of high-income nations, they are on the rise in many low- and middle-income nations (162). Ovarian cancer is a highly aggressive and frequently fatal form of gynecological cancer that originates in the ovaries. Lack of early symptoms contributes to its aggressiveness, resulting in late-stage diagnoses when the tumor has spread beyond the ovaries. The preponderance of ovarian cancers are epithelial ovarian carcinomas, and the most aggressive subtype is high-grade serous carcinoma. Due to the aggressive nature of ovarian cancer, prompt and accurate diagnosis, aggressive treatment strategies such as surgery and chemotherapy, and conducting research to identify effective targeted therapies are essential to improve patient outcomes (163).

Multiple studies have investigated the function of the lncRNA MEG3 in ovarian cancer. First, MEG3 was reported to regulate angiogenesis in ovarian cancer endothelial cells by sponging miRNA-376a and YBX1, with significantly lower expression in ovarian cancer endothelial cells than that in normal ovarian endothelial cells. Overexpressing MEG3 in ovarian cancer endothelial cells decreased tube formation through modulating the miR-376a/RASA1 axis (114) (**Figure 3**). Second, MEG3 inhibited the progression of ovarian cancer by sponging miR-30e-3p and regulating LAMA4 expression (115). In addition, MEG3 affected the epithelial-mesenchymal transition of ovarian cancer cells by sponging miR-219a-5p and regulating EGFR; low levels of MEG3 and miR-219a-5p were associated with a poor prognosis in patients with ovarian cancer (**Figure 3**). Knocking down MEG3 and miR-219a-5p in ovarian cancer cells increased the cellular viability, proliferation, invasion, and migration, and decreased apoptosis (**Table 2**) (129). Another study reported that MEG3 overexpression inhibited the cellular viability and invasion while promoting apoptosis in ovarian cancer through sponging miR-205-5p (130). A recent study in epithelial ovarian carcinoma (EOC) demonstrated that MEG-3 expression was significantly lower in EOC tissues compared to normal ovarian tissues. Receiver operating characteristic (ROC) analysis showed an AUC of 0.831, with high sensitivity (100%) and specificity (97.04%) in distinguishing malignant from normal tissues. Low MEG-3 expression correlated with advanced tumor stages, poor treatment response, and adverse prognostic factors, while high expression was associated with better survival outcomes (p < 0.001). These findings highlight MEG-3 as a promising biomarker for EOC diagnosis, prognosis, and personalized therapy (164). These studies collectively shed light on the diverse regulatory functions of MEG3 in ovarian cancer, indicating its potential role as a therapeutic target for this disease.

### 4.7 Endometrial cancer

Endometrial cancer is the most prevalent gynecological cancer in high-income countries, and its incidence is increasing worldwide. The increasing prevalence of obesity is the most significant underlying cause of endometrial cancer (165). The incidence and mortality rates of endometrial cancer have increased by 21% and >1000%, respectively, over the past two decades (166). Non-endometrioid subtypes of cancer, such as serous and clear cell carcinomas, have a greater propensity for invasion and metastasis. These subtypes are frequently diagnosed at a later stage, which renders treatment as more challenging. The identification of specific molecular and genetic alterations associated with aggressive endometrial cancer has led to advancements in targeted therapies. Accordingly, biomarkers such as *p53* mutations and DNA mismatch repair deficiencies may influence treatment decisions (167).

MEG3 was reported to be downregulated in highly invasive, sphere-forming, and TX-resistant endometrial cancer cell derivatives (168). In addition, MEG3 expression was substantially reduced in endometrial cancer tissues compared to that in adjacent normal tissues and normal endometrial cell lines, such as HEC-1A and KLE (132). Another study supported these findings by demonstrating that MEG3 expression was substantially lower in endometrial cancer samples compared to that in normal endometrial tissues (131). Notably, sustained overexpression of MEG3 in HEC-1B and Ishikawa cells induced apoptosis and decreased migration and invasion. In addition, MEG3 inhibited the tumorigenesis and progression of endometrial cancer by repressing the Notch signaling pathway (**Table 2**, **Figure 3**). Downregulation of MEG3 was associated with elevated levels of Notch1 and Hes1 proteins (132). In addition, MEG3 inhibited endometrial carcinoma cell proliferation, invasion, and metastasis, while inducing apoptosis and inhibiting the activation of the PI3K/m-TOR signaling pathway (131) (**Figure 3**). MEG3 could significantly suppress tumor growth as evidenced in tumor xenograft implantation in nude mice (131). Furthermore, MEG3 played a role in modulating PD-L1 expression in aggressive endometrial cancer cells; its expression was regulated by miR-216a (**Table 2**) (169). Together, these studies highlight the significant downregulation of MEG3 in endometrial cancer; its role in inhibiting cell proliferation, migration, and invasion; and its regulatory roles in modulating the Notch and PI3K pathways, thereby shedding light on potential therapeutic strategies for the treatment of endometrial cancer.

## 5 MEG3 as a diagnostic biomarker: challenges in clinical integration

The unique nature of lncRNAs, including their high specificity, and sensitivity in detecting cancer, makes MEG3 a promising diagnostic biomarker (170, 171). MEG3 can be detected in easily accessible samples such as blood or serum, reducing the need for invasive procedures like tissue biopsies (171–174). Additionally, its stability in these fluids and ability to identify early-stage malignancies highlight its value in improving diagnostic accuracy and patient outcomes (164, 171, 173). These advantages suggest that MEG3 could revolutionize cancer diagnostics. However, integrating MEG3 diagnostics into clinical workflows presents challenges including cost-effectiveness and technical feasibility. In terms of cost-effectiveness, the development of MEG3-based assays, such as qRT-PCR or sequencing techniques, may incur higher expenses compared to conventional diagnostic methods like immunohistochemistry.

Moreover, technical feasibility also poses a hurdle for implementing MEG3 diagnostics on a broad scale, requiring standardized protocols to address pre-analytical variability, such as differences in sample collection, storage, and handling, which can impact RNA stability and assay reliability (164). Addressing these challenges through the development of robust, simplified protocols and user-friendly testing kits could significantly enhance the practicality of MEG3 diagnostics. Furthermore, multicenter clinical trials are necessary to validate MEG3’s diagnostic performance and establish its superiority over existing biomarkers. Overcoming these hurdles will pave the way for MEG3 diagnostics to become a practical tool in routine clinical settings.

## 6 MEG3 in clinical applications

Based on the aforementioned, MEG3’s tumor-suppressor role in genitourinary cancers positions it as a promising biomarker for early diagnosis and prognosis, while also opening exciting avenues for translating laboratory findings into clinical applications. For instance, a recent study investigating the role of MEG3 in a homogenous series of advanced HGSOC patients demonstrated that exogenous MEG3 expression *in vitro* and *in vivo* could reverse the malignant phenotype of HGSOC cells, inhibiting cell proliferation, migration, invasion, and spheroid formation in mice (128). Moreover, elevated MEG3 expression was independently associated with better progression-free and overall survival in patients. These findings are likely mediated through positive regulation of the PTEN network, as PTEN loss is associated with poor prognosis and impaired therapeutic response in HGSOC. These preclinical results reinforce MEG3’s role as a tumor suppressor and highlight its potential as a promising therapeutic target (128).

Another preclinical study demonstrated that upregulation of MEG3 in prostate cancer (PCa) cell lines induced apoptosis and G0/G1 phase arrest, to inhibit the expression of Bcl-2 and Cyclin D1. *In vivo*, MEG3 overexpression in PC3 cells resulted in significantly reduced tumor weight and volume in mice, reinforcing its tumor-suppressive properties. Overall, these results highlight the therapeutic potential of MEG3, opening up opportunities for its clinical application in prostate cancer treatment (60).

A new study demonstrates that MEG3 is downregulated in PCa tissues, with lower expression levels correlating with poorer prognosis. This study revealed that overexpression of MEG3 inhibited PCa cell proliferation and promoted apoptosis (175). Mechanistically, MEG3 acts as a ceRNA by binding miR-9-5p, thereby alleviating its repressive effect on NDRG1, a protein that regulates cell proliferation and apoptosis. Furthermore, preclinical studies have demonstrated that the upregulation of MEG3 significantly inhibits tumor growth in animal models. All of these findings support the therapeutic promise of MEG3 in PCa treatment (175).

Furthermore, another study MEG3 plays a critical role in bladder cancer by modulating miR-494 and the tumor suppressor gene PTEN. *In vitro* and *in vivo* assays have revealed that MEG3 inhibits the expression of miR-494 in bladder cancer cells. Thus, restoration of MEG3 levels holds therapeutic promise for the treatment of bladder cancer (109). Collectively, these findings have propelled MEG3 into the spotlight as a target for therapeutic interventions, encouraging the need for well-designed clinical trials to evaluate its efficacy and safety in patients with genitourinary cancers.

However, while MEG3’s tumor suppressive functions are well-documented in genitourinary cancers, its potential as a standalone therapy remains underexplored and requires further investigation. Current data suggest that MEG3 may be more effective as an adjuvant therapy rather than a primary treatment. For instance, in bladder and cervical cancers, MEG3 overexpression has been shown to improve the efficacy of chemotherapeutic agents like cisplatin by promoting apoptosis and mitigating cellular invasion and survival pathways (121, 126). This synergistic effect highlights MEG3’s potential to enhance chemosensitivity and improve outcomes in advanced or treatment-resistant cancers.

## 7 Barriers for advancing MEG3 in clinical therapy and future direction

Despite these promising findings, significant challenges remain in MEG3-based therapies into clinical practice. A primary barrier is the effective delivery of MEG3 to target tissues. RNA-based therapeutics, including antisense oligonucleotides (ASOs) and small interfering RNAs (siRNAs), those aimed at modulating MEG3 expression, often encounter issues such as rapid degradation by nucleases, poor cellular uptake, and limited bioavailability at the tumor site (176, 177). These limitations hinder the precise delivery of MEG3-targeting agents to their intended sites of action. Delivery systems such as viral vectors, lipid nanoparticles, and exosome-based platforms offer potential solutions. However, each of these systems presents unique challenges regarding efficiency, safety, and specificity (176, 177). For instance, while lipid nanoparticles can enhance cellular uptake, they may also provoke immune responses that could compromise their therapeutic efficacy (177). Therefore, further research is essential to optimize these delivery mechanisms, ensuring that therapeutic agents, such as MEG3 effectively reach their intended targets within the tumor microenvironment.

Another major concern is the potential for off-target effects. As a ceRNA, MEG3 interacts with various miRNAs and downstream pathways, which could lead to adverse effects if misregulated, leading to undesirable unintended consequences, such as toxicity (177). To mitigate this, future studies should focus on engineering MEG3-based therapies to maximize specificity and minimize unintended interactions. High-throughput screening approaches could play a pivotal role in mitigating off-target effects, thus enhancing the safety of MEG3-based interventions (177).

While preclinical models may demonstrate safety, human responses can vary considerably, as evidenced by immune-related toxicities observed in some clinical trials of RNA-based therapeutics. These toxicities highlight the importance of rigorous validation and meticulous testing in preclinical models and patient cohorts to minimize adverse effects and improve therapeutic outcomes. Finally, while MEG3-centered therapeutic strategies offer significant promise in cancer treatment, addressing the limitations related to delivery mechanisms and off-target effects is essential. Continued research and development efforts are necessary to overcome these challenges, ultimately enhancing the therapeutic benefits of targeting MEG3 in cancer therapy.

## 8 Conclusion

This review provides a comprehensive overview of the emerging roles of the tumor suppressor MEG3 in the genitourinary cancers, positioning it as a promising therapeutic target. MEG3’s potential for improving diagnostics and therapeutic strategies offers exciting opportunities for advancing treatment options in these cancers. However, significant challenges remain in translating MEG3-based therapies into clinical practice, particularly in optimizing delivery mechanisms and minimizing off-target effects.

Future research should focus on refining MEG3-based strategies, exploring synergistic effects with conventional treatments like chemotherapy and immunotherapy, and addressing the logistical challenges associated with clinical implementation. By overcoming these barriers, MEG3 could significantly improve outcomes for patients with genitourinary cancers and pave the way for its integration into precision oncology.
